# Enhancing the Care of Patients With Bone and Joint Infections Through Educational Interventions

**DOI:** 10.1093/ofid/ofaf552

**Published:** 2025-09-02

**Authors:** Neel B Shah, Jessica Seidelman, Katherine Belden, Elizabeth Thottacherry, Allison Lastinger, Priya Nori, Ronald M Beaulieu, Don Bambino Geno Tai

**Affiliations:** Division of Infectious Diseases, Department of Internal Medicine, University of Pittsburgh Medical Center, Pittsburgh, Pennsylvania, USA; Division of Infectious Diseases, Department of Internal Medicine, Duke University, Durham, North Carolina, USA; Division of Infectious Diseases, Department of Internal Medicine, Thomas Jefferson University Hospital, Philadelphia, Pennsylvania, USA; Division of Infectious Diseases and Geographic Medicine, Department of Medicine, Stanford University School of Medicine, Palo Alto, California, USA; Division of Infectious Diseases, Department of Medicine, West Virginia University, Morgantown, West Virginia, USA; Division of Infectious Diseases, Department of Medicine, Montefiore Health System, Albert Einstein College of Medicine, Bronx, New York, USA; Division of Infectious Diseases and Tropical Medicine, Georgetown University Hospital, Washington, District of Columbia, USA; Division of Infectious Diseases and International Medicine, Department of Medicine, University of Minnesota Medical School, Minneapolis, Minnesota, USA

**Keywords:** bone, education, infections, joint, musculoskeletal

## Abstract

Musculoskeletal infections represent a significant proportion of the clinical workload for infectious diseases (ID) providers, often presenting complex diagnostic and therapeutic challenges. Many providers may feel inadequately prepared to manage these conditions, which require a nuanced understanding of microbiology, musculoskeletal anatomy, surgical intervention, and orthopedic hardware. We implemented a pair of educational interventions including an online library of high-yield musculoskeletal ID literature and an educational podcast.

Musculoskeletal infectious diseases (MSK-ID) make up a significant proportion of infectious diseases (ID) practice, with a rising incidence of bone and joint infections across different populations. This rise is attributed to an aging population, an increase in orthopedic procedures, an increasing prevalence of comorbidities, and immunosuppression [1, 2]. Furthermore, the increasing use of orthopedic implants and the spread of antibiotic-resistant organisms have heightened the complexity of these infectious syndromes [3]. Certain orthopedic-related infections such as periprosthetic joint infection (PJI) carry a 5-year mortality risk comparable to certain malignancies, and the cost per patient for orthopedic-related surgical site infections can exceed $100 000 [4, 5]. Hence, such infections have the potential to cause significant morbidity and mortality, and incur substantial financial cost within the healthcare system [6]. Diabetes-related foot infections (DFIs) affect roughly 40% of foot ulcers and are associated with poor outcomes. Among hospitalized patients with DFIs, up to half require amputation, and the 5-year mortality rate after a major amputation exceeds 50% [7]. Regarding postoperative spinal implant infections, the treatment success rate of debridement and implant retention is dismal at 71%–73%, and the role of suppressive antibiotic therapy is unknown [8].

Despite the immense challenges associated with bone and joint infections, MSK-ID remains underrepresented in medical education. There is a lack of structured and comprehensive medical training with regard to these infections, and only a limited number of advanced fellowship programs are available within the United States focusing on MSK-ID [9]. The standard ID fellowship curriculum places more emphasis on general ID topics, without a specialized focus on musculoskeletal infections [7]. Consequently, ID clinicians may feel inadequately prepared to manage these conditions after completion of their fellowship training, as they often require a nuanced understanding of MSK-ID pathophysiology such as biofilm formation, advancing surgical techniques, and orthopedic implants.

Although there is an abundance of literature regarding bone and joint infections, findings often conflict, making it difficult to ascertain what studies are more robust and higher in yield. Publications on the same subject often vary in methodological quality and applicability to clinical practice, making it difficult for clinicians to identify studies that are most impactful for clinical care [10]. This lack of clarity in the literature, combined with limited exposure provided during medical training, can result in suboptimal management, impacting treatment outcomes. Despite these shortcomings, clear benefits in multidisciplinary management of bone and joint infections by MSK-ID–trained physicians and orthopedic specialists have been established, reinforcing an urgent need for such expertise in the medical field [7, 9]. In order to address these challenges, a multimodal approach is required in optimizing MSK-ID education.

## AN ORTHO ID ONLINE LIBRARY

To better address the disjointed information regarding bone and joint infections within the literature, a group of ID physicians within the Musculoskeletal Infection Society (MSIS) gathered to create an online Ortho ID library (www.orthoidlibrary.com), a centralized, easy-to-navigate library platform aimed at providing a comprehensive collection of peer-reviewed articles on select topics. We focused on 8 high-yield topics, chosen by consensus and identified for their educational and research significance. These include PJI, DFIs, native vertebral osteomyelitis, spinal implant infections, fracture-related infections, native septic arthritis, non-implant-associated osteomyelitis, and sacral osteomyelitis.

Studies with a higher level of evidence (randomized controlled trials, meta-analyses, large observational studies) were prioritized. We focused on literature published from 2013 onward to ensure that the selected articles reflect contemporary research and practice. Narrative reviews and society clinical practice guidelines that were of notable educational benefit were also included. Low-level evidence (case reports/series, small retrospective studies) without robust methodology were excluded. Some articles were summarized using ChatGPT (OpenAI, San Francisco, California, USA). Librarians used a standardized prompt to highlight key aspects of each article. One example of a prompt we used is: “You are an expert in evidence-based medicine. Review the uploaded article and summarize the key question, main findings, and clinical implications. Identify the strengths and limitations of the study. Assess the level of evidence using the GRADE system (very low, low, moderate, high) and provide the rationale for your rating.”

The output summarized the main question(s), findings, clinical implications, strengths, and weaknesses, and included a Grading of Recommendations Assessment, Development, and Evaluation (GRADE)–based evidence assessment, offering a perspective distinct from the article's abstract. After review by the librarian, this synopsis was added as a supplement to the article. These procedures are currently in a trial phase and have not yet been standardized.

There were 2990 sessions in 2024, and the Ortho ID library was accessed by 2053 active users from 75 countries. Maintenance has been conducted by the group who continue to add articles and synopses, which are reviewed by the larger team. Currently, all members responsible for its upkeep are volunteers who balance this work with other academic research and educational commitments, making it challenging to keep the content consistently up to date. The curation process is manual rather than a formal systematic review, which means some important studies may be unintentionally overlooked. To help address this, we introduced a feedback form that allows library users to recommend journal articles for inclusion, suggest edits to synopses, and share general feedback, aiming to capture as much high-yield literature as possible. Other challenges for the library include the ongoing cost of website hosting and occasional inadvertent blocking by university and institutional information technology systems.

## *THE JOINT APPROACH*: A PODCAST

Medical education in ID has evolved to include new ways of engaging learners, including the use of podcasts. *The Joint Approach* podcast, launched in August 2023, aims to bridge communication and understanding between medical and surgical disciplines. Co-hosted by an ID physician and an orthopedic surgeon, the podcast explores the “joint” challenges between the 2 specialties in the diagnosis and treatment of patients with musculoskeletal infections. Episodes explore existing data and facilitate dialogue between the specialties, offering valuable insights into navigating complex clinical cases.

To date, there have been a total of 11 177 all-time listens across 46 episodes. The topics discussed on the podcast are carefully chosen by the hosts based on conversations with peers in the MSK-ID community and recent, impactful publications. Guests are selected with equal deliberation, typically featuring an ID physician and an orthopedic surgeon. Top episodes to date include “Systemic Antibiotics” with 558 listens, “Diabetic Foot Infections (Part 1)” with 480 listens, and “Biofilm (Part 1)” with 432 listens. The podcast strives to present diverse perspectives, and the primary criterion for guest selection is expertise in the episode's subject matter. The podcast effectively reaches healthcare professionals seeking to deepen their understanding of musculoskeletal infections. We believe that *The Joint Approach* podcast fosters interdisciplinary dialogue and leverages innovative medical education platforms, playing a pivotal role in advancing knowledge in this critical field.

## FUTURE STEPS: EDUCATION AS AN ESSENTIAL TOOL

Great strides in the MSK-ID specialty have been made in the past decade, with work to standardize the diagnosis of PJI, patient selection for less invasive surgery, and expanded use of oral antimicrobials [11–13]. Raising awareness of the work being done in MSK-ID is critical to closing knowledge gaps and improving patient outcomes. The MSIS and the International Consensus Meeting (ICM) on Musculoskeletal Infection serve as essential educational forums for advancing and promoting the MSK-ID specialty. Expanding musculoskeletal infection education within medical school and residency curriculums is another key step toward addressing these gaps and promoting best practices. It may also be worth considering the development of a standardized curriculum, collaboratively designed by leading experts, to serve as a model for other institutions aiming to enhance their MSK-ID training.

Importantly, society resources and guidelines are critical in providing evidence-based recommendations to drive best practices. The most recent Infectious Diseases Society of America (IDSA) practice guidelines related to MSK-ID, focused on the management of DFIs, were published in 2023—a sharp contrast to the guidelines for treating native vertebral osteomyelitis, which were published a decade ago. The MSIS, ICM, and European Bone and Joint Infection Society have all published guidance on the diagnosis of PJI and definition of treatment success [14–17], although IDSA has not done so since 2012. Last, further incorporation and early adoption of artificial intelligence and large language models into medical education curriculum will be instrumental in enhancing MSK-ID education, along with other fields of medicine, within medical schools and residencies.

We hope that the above interventions serve as a starting point for increasing MSK-ID awareness and provide learners with easy access to high-yield, impactful educational material that can guide the complex decision-making associated with these infections, allowing for them to optimize management and potentially standardize care in the future.
